# *In vitro* Activities of Nemonoxacin and Other Antimicrobial Agents Against Human *Mycoplasma* and *Ureaplasmas* Isolates and Their Defined Resistance Mechanisms

**DOI:** 10.3389/fmicb.2019.01890

**Published:** 2019-08-13

**Authors:** Na Wang, Wancheng Liu, Yunheng Zhou, Yang Liu

**Affiliations:** ^1^Institute of Antibiotics, Huashan Hospital, Fudan University, Shanghai, China; ^2^Key Laboratory of Clinical Pharmacology of Antibiotics, Ministry of Health, Fudan University, Shanghai, China; ^3^Suzhou Institute of Systems Medicine, Chinese Academy of Medical Sciences & Peking Union Medical College, Suzhou, China; ^4^Department of Clinical Laboratory, Shanghai Corps Hospital, Chinese People’s Armed Police Forces, Shanghai, China

**Keywords:** nemonoxacin, *Mycoplasma*, *Ureaplasma*, antimicrobial resistance, MICs

## Abstract

Nemonoxacin, a newly developed non-fluorinated quinolone (NFQ), selectively inhibits bacterial DNA topoisomerase activity. However, its activities against *Mycoplasmas* have rarely been studied to date. Herein, the activities of nemonoxacin were evaluated against clinical isolates of 50 *Mycoplasma pneumoniae*, 20 *Mycoplasma hominis*, and 77 *Ureaplasma* spp., and they were compared to fluoroquinolones, tetracyclines, and macrolides. Nemonoxacin MICs (μg/ml) ranged from 0.03 to 0.25 for *M. pneumoniae*, 0.25 to 8 for *M. hominis*, and 0.06 to >16 for *Ureaplasma* spp., and all of the ranges are similar to those of fluoroquinolones. The activity of nemonoxacin against *Mycoplasmas* was not affected by resistance to macrolides in the strains tested, but it seems to have the same resistant mechanism as fluoroquinolones. In addition, minimum bactericidal concentrations (MBC) of nemonoxacin to *M. pneumoniae* were within two dilutions of the MIC values, indicating a bactericidal effect on *M. pneumoniae*. Nemonoxacin merits further study for treating infections caused by these organisms.

## Introduction

Nemonoxacin, a newly developed non-fluorinated quinolone (NFQ), has showed broad-spectrum activity against Gram-positive, Gram-negative, and atypical pathogens *in vitro* and is generally safe and well tolerated *in vivo* (Chung et al., 2010). It has rapid oral absorption with a peak serum concentration (C_*max*_) attained in 1–2 h and an elimination half-life of 12.83–18.56 h. The binding of nemonoxacin to serum proteins is approximately 16%, which is lower than any currently existing quinolone on the market (Lai et al., 2014). Nemonoxacin has more than 95% bioavailability, which is similar to levofloxacin and moxifloxacin (File, 2010; Wilson and Macklin-Doherty, 2012). In October 2016, the China Food and Drug Administration approved nemonoxacin malate capsules as a clinical drug for the treatment of community-acquired pneumonia (CAP) in adults. Although clinical trials demonstrated that nemonoxacin had favorable bacteriological success against atypical pathogens, there are no available data on the activity of nemonoxacin against *Mycoplasma pneumoniae*, *Ureaplasma* spp., and *Mycoplasma hominis*, which necessitates exploration (van Rensburg et al., 2010; Huang et al., 2015).

*Mycoplasma pneumoniae* is a common microorganism found in respiratory tract infections in children and adults and is also responsible for many extrapulmonary manifestations, such as dermatological disorders, central nervous system diseases, and thrombotic thrombocytopenic purpura, which can range in severity from mild to life-threatening (Waites et al., 2017b). *Ureaplasma* spp. and *M. hominis*, are frequently found in human urogenital symptomatic patients and healthy individuals (Horner et al., 2018). *Ureaplasma* spp. can be grouped into *Ureaplasma parvum* (biovar 1) and *Ureaplasma urealyticum* (biovar 2), whose DNA homology is less than 60%. *U. parvum* is more common than *U. urealyticum*, when isolated from clinical specimens, but both biovars may occur simultaneously in some patients (Fanrong et al., 1999; Waites et al., 2005). In men, *M. hominis* and *U. parvum* are not evidently related to diseases, but *U. urealyticum* is associated with non-gonococcal urethritis cases, but there is no link to infertility. In women, there is no adequate evidence that these three organisms can result in cervicitis, urethritis, inflammatory vulvovaginitis, or infertility (Horner et al., 2018). In addition to urogenital diseases, *Ureaplasma* spp. is also associated with adverse pregnancy outcomes and newborn diseases, such as chronic lung disease and retinopathy of prematurity (Kokkayil and Dhawan, 2015).

Macrolides, tetracyclines, and fluoroquinolones are three common antibiotic families against *M. pneumoniae, Ureaplasma* spp., and *M. hominis*. Acquired macrolide resistance to *M. pneumoniae* strains in clinical settings has emerged worldwide and is currently complicating treatment, especially in Asia (Waites et al., 2014). Mutations at various positions in 23S rRNA and insertions or deletions in ribosomal proteins L4 and L22 can cause macrolide resistance of *M. pneumoniae* (Okazaki et al., 2001; Matsuoka et al., 2004). An increasing prevalence of tetracycline and fluoroquinolone resistance of *Ureaplasma* spp. and *M. hominis* has been reported in Europe and North America, whereas it is rare in China (Xiao et al., 2012; Beeton and Spiller, 2016; Meygret et al., 2018). Additionally, *Ureaplasma* spp. resistant rates against macrolides are very low (Schneider et al., 2015; Fernandez et al., 2016). Acquired high-level tetracyclines resistance is related to the presence of the *tet*(M) gene, while acquired fluoroquinolones resistance is associated with mutations in the quinolone resistance-determining regions (QRDRs) of the *gyrA*, *gyrB*, *parC*, and *parE* genes (Bebear et al., 2003; Beeton and Spiller, 2016). Here, the aim of this study was to assess the susceptibility of *M. pneumoniae, Ureaplasma* spp., and *M. hominis* to *in vitro* nemonoxacin and other drugs, including 16-membered macrolide. In addition, the resistance mechanisms of resistant isolates were also evaluated.

## Materials and Methods

### *Mycoplasmas* Clinical Strains Culture

1. *Mycoplasma pneumoniae:* Fifty *M. pneumoniae* clinical strains were originally obtained from Shanghai Children’s Hospital between November 2017 and August 2018 and were stored in the strain bank at the Institute of Antibiotics, Huashan Hospital at −80°C. *M. pneumoniae* culturing was performed as described previously (Dorigo-Zetsma et al., 1999). All of the strains were identified by colony morphology and PCR assays. PCR amplification of the 16S rRNA genes was performed to identify *M. pneumoniae* using 5′-GCCACCCTCGGGGGCAGTCAG-3′ and 5′-GAGTCGGGATTCCCCGCGGAGG-3′ primers as previously described (Ieven et al., 1996).
2. *Mycoplasma hominis:* Twenty *M. hominis* clinical strains were originally obtained from Shanghai Corps Hospital of Chinese People’s Armed Police between 2013 and 2018 and stored in the strain bank at the Institute of Antibiotics, Huashan Hospital at –80°C. *M. hominis* culturing was performed as described previously (Novosad et al., 2017). All strains were identified by colony morphology.
3. *Ureaplasma* spp.: Seventy-seven *Ureaplasma* spp. clinical strains were originally obtained from Shanghai Corps Hospital of Chinese People’s Armed Police between 2013 and 2018 and stored in the strain bank at the Institute of Antibiotics, Huashan Hospital at –80°C. *Ureaplasma* spp. culturing was performed as described previously (Xiao et al., 2010). All strains were identified by colony morphology and PCR assays. Confirmation of *Ureaplasma* spp. was determined by amplification of the Ureaplasma-specific urease gene (a 430-bp DNA product) using Blanchard et al. (1993) U4 and U5 primers. After confirmation, isolates were further separated into species based on PCR primers (UM-1), as published by Teng et al. (1994). A region of the multiple-banded antigen (MBA) was amplified and sequenced. The UM-1 primer sets for *U. parvum* yielded a 403-bp product, and there was a 448-bp product for *U. urealyticum*.

The study was approved by the Ethics Committee of Huashan Hospital, Fudan University, China. All strains we used in this study were obtained from biological samples from the strain bank at the Institute of Antibiotics, Huashan Hospital, Shanghai. These samples were obtained during routine clinical testing, and then they were stored in the strain bank. The ethics committee of Huashan Hospital authorized our study, and written informed consent was not required. This study did not harm the rights, benefits and health of the subjects, and the privacy and personal identity information of the subjects has been and will be protected.

All reference strains, including ATCC 29342 (*M. pneumoniae*), ATCC 23114 (*M. hominis*), ATCC33699 (*U. urealyticum*), and ATCC27815 (*U. parvum*) were purchased from China’s Antubio (603658.SH).

### Test for *Mycoplasmas* Sensitivity to Antibiotics

In this study, 11 antimicrobials were used, including: Nemonoxacin (manufactured by Zhejiang Medicine Co., Ltd., Zhejiang, China), macrolides (erythromycin, roxithromycin, azithromycin, and josamycin), tetracyclines (tetracycline, minocycline, and doxycycline), and fluoroquinolones (ciprofloxacin, levofloxacin, and moxifloxacin) (Dalian Meilun Biotech Co., China). All agents were obtained in powdered form of known purity and diluted according to their respective manufacturer’s instructions. The broth microdilution method was performed and the minimal inhibitory concentration (MIC) was defined according to Clinical and Laboratory Standards Institutes (Waites et al., 2011).

The minimum bactericidal concentrations (MBCs) for nemonoxacin was determined as previously described (Waites et al., 2017a), which included 11 *M. pneumoniae* isolates, 5 *M. hominis* isolates, and 5 *Ureaplasma* spp. isolates. The positive and negative controls consisted of tetracycline (bacteriostatic) and moxifloxacin (bactericidal), respectively. When the MBC was ≥3 dilutions (eightfold) greater than the MIC, the drug was considered bacteriostatic. MBCs that were ≤2 dilutions (fourfold) greater than the MIC were considered bactericidal.

### Mutation Point Detection

For *M. pneumoniae*, the total DNA was extracted by manual nucleic acid extraction (Qiagen QI Amp DNA Mini Kit, Valencia, CA, United States). PCR assays for 23S rRNA were performed to amplify and sequence the full length of the 23S rRNA fragment, as described previously (Liu et al., 2009). In addition, the L4 and L22 ribosomal protein genes were amplified and sequenced (Pereyre et al., 2004).

For *M. hominis*, antibiotic susceptibility testing of the *M. hominis* strains have been previously described (Bebear et al., 1997). Total DNA of resistant strains was used as a template in a PCR to amplify the QRDRs of the *gyrA*, *gyrB*, *parC*, and *parE* (Bébéar et al., 1998).

For *Ureaplasma* spp., DNA was extracted by manual nucleic acid extraction (Qiagen QI Amp DNA Mini Kit, Valencia, CA, United States). For *U. parvum*, primers for gyrA-1, gyrA-2, gyrB-3, gyrB-4, parC-5, parC-6, parE-7, and parE-8 were used to amplify the *gyrA*, *gyrB*, *parC*, and *parE* genes, respectively. For *U. urealyticum*, primers for the *gyrA*, *parC*, and *parE* genes were the same as the primers used for *U. parvum*, whereas primers for gyrB-F-UuUp and gyrB-R-UuUp were designed to amplify *gyrB*, as described previously (Meygret et al., 2018). Primers for TetMF and TetMR were used to verify the existence of *tet*(M) in tetracycline-resistant strains. All gene sites and protein residues were numbered according to *M. pneumonia*, *M. hominis*, or *Ureaplasma* spp.

## Results

### Nemonoxacin Was Active Against *M. pneumoniae*

Here, we detected the nemonoxacin MIC in 50 *M. pneumoniae* isolates, which suggested that nemonoxacin was active *in vitro* against all 50 clinical isolates of *M. pneumoniae*. Nemonoxacin and moxifloxacin had the lowest MIC_50_ and MIC_90_ values (0.125 and 0.25 μg/ml, respectively) among all 11 drugs tested against *M. pneumoniae*, and their dilutions were twofold lower than those of the tetracyclines and levofloxacin, with all nemonoxacin MICs of ≤0.25 μg/ml (Table 1). We found that 46 of the 50 isolates were macrolides-resistant organisms and had A to G mutation at position 2,063 in their 23S rRNA gene compared with the *M. pneumoniae* reference strain. No mutations in ribosomal proteins L4 and L22 were found. These organisms had elevated MICs for erythromycin, roxithromycin, and azithromycin, ranging from 4 to >128 μg/ml, while the nemonoxacin MICs were 0.06 to 0.25 μg/ml. Azithromycin possessed lower MIC_50_ and MIC_90_ levels (8 and 32 μg/mL, respectively) than those of erythromycin and roxithromycin. Additionally, the 16-member macrolide josamycin had lower MICs than the 14- and 15-member macrolides. All 50 *M. pneumoniae* isolates were susceptible to the tetracyclines (tetracycline, doxycycline, and minocycline) and fluoroquinolones (ciprofloxacin, levofloxacin, and moxifloxacin) we tested. An exhaustive MIC distribution of the 11 antibiotics against *M. pneumoniae* is shown in Supplementary Table 1. Taken together, these results suggest that nemonoxacin and moxifloxacin were active against *M. pneumoniae* and had lower MIC levels than other antibiotics.

**TABLE 1:** Minimal inhibitory concentration summary of nemonoxacin and comparator agents against *M. pneumoniae*.

**Isolates and reference strains**	***M. pneumoniae* (*n* = 50)^*a*^**			**ATCC 29342**	**MIC (μg/ml)^*b*^**	
			
	**MIC range**	**MIC_50_**	**MIC_90_**	**MIC**	**S**	**R**
**Antimicrobial agents (μg/ml)**						
Nemonoxacin	0.03 to 0.25	0.125	0.25	0.125	−	−
Moxifloxacin	0.015 to 0.25	0.125	0.25	0.25	≤0.05	−
Levofloxacin	0.03 to 1	0.5	0.5	0.25	≤1	−
Ciprofloxacin	0.03 to 2	1	2	0.25	−	−
Tetracycline	0.06 to 2	0.25	0.5	0.5	≤2	−
Minocycline	0.03 to 2	0.25	0.5	0.5	−	−
Doxycycline	0.015 to 2	0.5	0.5	0.25	−	−
Erythromycin	0.0075 to >128	>128	>128	0.0075	≤0.5	≥1
Roxithromycin	≤0.00375 to >128	64	>128	0.03	−	−
Azithromycin	≤0.00375 to 64	8	32	0.0075	≤0.5	≥1
Josamycine	≤0.00375 to 32	2	4	0.03	−	−

*^*a*^Includes 48 *M. pneumoniae* type I and 2 *M. pneumoniae* type II organisms, and 46 macrolide-resistant organisms; ^*b*^Interpretive criteria from CLSI M43-A. S, susceptible; R, resistant.*

### The Activity of Nemonoxacin Against *M. hominis* Is Similar to That of Fluoroquinolones

The nemonoxacin MICs against 20 *M. hominis* clinical strains isolated from urogenital specimens ranged from 0.25 to 8 μg/ml and had an MIC_50_ of 2 μg/ml, making nemonoxacin less active *in vitro* than the tested tetracyclines (Table 2). Due to the intrinsic resistance of *M. hominis* to 14- and 15-membered macrolides, the MICs of erythromycin, roxithromycin, and azithromycin were >128 μg/ml. However, josamycin, as a 16-membered macrolide, had lower MIC_50_ and MIC_90_ levels than the fluoroquinolones and nemonoxacin, but the levels were higher than those of the tetracyclines. In these drugs, the resistance breakpoints of josamycin, ciprofloxacin, doxycycline, and minocycline were not defined. A MIC distribution of the 11 antibiotics against *M. hominis* is shown in Supplementary Table 2. No tetracycline-resistant organisms were found, but 16 out of the 20 *M. hominis* isolates were fluoroquinolone-resistant organisms. The tetracyclines, especially doxycycline and minocycline, had the lowest MIC_50_ and MIC_90_ values among the 11 drugs tested against *M. hominis*.

**TABLE 2:** Minimal inhibitory concentration summary of nemonoxacin and comparator agents against *M. hominis*.

**Isolates and reference strains**	***M. hominis* (*n* = 20)^*a*^**			**ATCC 23114**	**MIC (μg/ml)^*b*^**	
			
	**Range**	**MIC_50_**	**MIC_90_**	**MIC**	**S**	**R**
**Antimicrobial agents (μg/ml)**						
Nemonoxacin	0.25 to 8	2	8	0.25	−	−
Moxifloxacin	0.06 to 8	0.5	8	0.125	≤0.25	≥0.5
Levofloxacin	0.25 to 16	4	8	0.5	≤1	≥2
Ciprofloxacin	0.5 to >16	4	16	1	−	−
Tetracycline	0.03 to 0.125	0.06	0.06	0.06	≤4	≥8
Minocycline	≤0.0075 to 0.015	0.0075	0.0075	≤0.0075	−	−
Doxycycline	≤0.0075 to 0.03	0.0075	0.0075	≤0.0075	−	−
Erythromycin	>128	>128	>128	>128	−	−
Roxithromycin	>128	>128	>128	>128	−	−
Azithromycin	64 to >128	>128	>128	>128	−	−
Josamycine	0.125 to 1	0.5	0.5	0.5	−	−

*^*a*^Includes 16 fluoroquinolone-resistant organisms; ^*b*^Interpretive criteria from CLSI M43-A. S, susceptible; R, resistant.*

By sequencing *gyrA, gyrB, parC*, and *parE*, some gene point mutations and amino acid changes were found (Table 3). These mutations were mainly located at position 458 (C-T) and 606 (T-C) for *gyrA*, 272 (G-T) for *parC*, which lead to a substitution of Ser-153 by Leu in the gyrA protein, and Ser-91 by Ile in the parC protein. Only one strain had a gene point mutation and amino acid changes in the parE protein (Pro445Ser, C1333T). In *gyrA* and *parC*, there are many synonymous mutations apart from only one point mutation corresponding to one amino acid substitution. Collectively, these results suggest that quinolone resistance determining region mutations, such as gyrA and parC, may be related to elevated MICs of nemonoxacin to *M. hominis*.

**TABLE 3:** Gene point mutation and amino acid changes in GyrA, GyrB, ParC, and ParE in the Mh clinical isolates.

**Isolate number**	**Antibiotic resistance MIC (μg/ml)**				**Mutation position^a^**			
		
	**Nemonoxacin**	**Moxifloxacin**	**Levofloxacin**	**Ciprofloxacin**	**GyrA**	**GyrB**	**ParC**	**ParE**
Mh1	2	0.06	4	2	−	−	Ser91Ile (A207G, **G272T**, 285G)	−
Mh2	4	2	8	4	Ser153Leu (**C458T**, T513A, C528T, T606C)	−	Ser91Ile (**G272T**, A285G, C369T)	−
Mh3	8	8	16	>16	Ser153Leu (**C458T**, T513A, C528T, T606C)	−	Ser91Ile (**G272T**, A431G)	−
Mh4	8	8	8	>16	Ser153Leu (G408A, **C458T**, C528T, C594T, C660T)	−	Ser91Ile (**G272T**, T261A, A288G, C369T, C375T, C489G, G504A, T549C)	−
Mh5	2	0.125	4	2	−	−	Ser91Ile (**G272T**, A207G, A285G, C369T, C375T)	−
Mh6	4	0.125	8	4	−	−	Ser91Ile (**G272T**, T261A, A288G, C369T, C375T, C489G, G504A, T549C)	−
Mh7	2	0.125	8	4	−	−	Ser91Ile (**G272T**, T261A, A288G, C369T, C375T, C489G, T549C)	−
Mh8	2	0.06	4	2	−	−	Ser91Ile (**G272T**, A207G, A285G, A431G)	−
Mh9	8	8	8	16	Ser153Leu (**C458T**, C528T, C594T, C660T)	−	Ser91Ile (**G272T**, A431G)	−
Mh10	4	8	8	8	Ser153Leu (T363C, T369C, G408A, **C458T**, C528T, C594T, C660T)	−	Ser91Ile (**G272T**, A207G, A288G, A431G, T549C)	−
Mh11	1	2	4	8	Ser153Leu (G408A, **C458T**, T606C, G627A)	−	Ser91Ile (**G272T**, T261A, A288G, C369T, C375T, C489G, T549C)	−
Mh12	2	4	8	16	−	−	Ser91Ile (**G272T,** A431G)	−
Mh13	4	4	4	8	Ser153Leu (G408A, **C458T**, T606C, G627A)	−	Ser91Ile (**G272T**, T261A, A288G, C369T, C375T, C489G, T549C)	−
Mh14	2	4	4	8	Ser153Leu (A396G, G408A, A432G, **C458T**, C528T, A540T, C555T, C660T, C684T)	−	Ser91Ile (A207G, **G272T**, A285G, C369T, C375T)	−
Mh15	2	4	4	16	Ser153Leu (**C458T**, T513A, C527T, T606C)	−	Ser91Ile (**G272T**, A431G, G460A)	−
Mh16	1	0.5	2	8	Ser153Leu (T387C, A432G, T450C, **C458T**, C528T, C549T, A552T, C555T, T564A, C684T)	−	Ser91Ile (A207G, **G272T**, A285G, C369T, C375T)	Pro445Ser (**C1333T**)
Mh17	0.5	0.25	0.25	1	−	−	−	−
Mh18	0.25	0.06	0.25	0.5	−	−	−	−
Mh19	0.25	0.125	0.25	1	−	−	−	−
Mh20	0.25	0.125	0.5	1	−	−	−	−

*^*a*^The information presented in parenthesis are mutations of the corresponding genes. Bold fonts are in correspondence with amino acid substitutions particularly. Others are synonymous mutations.*

### MICs of Nemonoxacin for *Ureaplasma* spp.

Of the 77 *Ureaplasma* spp., 64 isolates were *U. parvum* (83.12%), and 13 were *U. urealyticum* (16.88%). All isolates were sensitive to erythromycin and had lower MIC_50_ and MIC_90_ values for azithromycin, roxithromycin, and josamycine compared with the fluoroquinolones. Only one tetracycline resistant isolate (tetracycline MIC > 16 μg/ml) with *tet*(M) was found, while the *tet*(M) element was not detected in the other tetracycline susceptible isolates.

In all the *U. parvum* isolates, the nemonoxacin MICs (MIC_50_ = 1 μg/ml, MIC_90_ = 2 μg/ml) were higher than any of the other comparator agents, except levofloxacin (Table 4). There were 2 *U. parvum* strains resistant to fluoroquinolones, whose nemonoxacin MICs were 4 and 8 μg/ml. One mutation in gryB (C1384T, Pro153Ser) was found in only one *U. parvum* resistant isolate (levofloxacin MIC = 16 μg/ml, moxifloxacin MIC = 4 μg/ml). Another *U. parvum* isolate (levofloxacin MIC = 4 μg/ml) showed fluoroquinolone resistance, but it still retained all wild-type sequences.

**TABLE 4:** Minimal inhibitory concentration summary for nemonoxacin and the comparator agents.

**Isolates and reference strains**	***Ureaplasma* spp. (*n* = 77)**			**ATCC 33175**	**MIC (μg/ml)^c^**	
		
	**Range**	**MIC 50**	**MIC 90**	**MIC**	**S**	**R**
**Antimicrobial agents (μg/ml)^a^**						
Nemonoxacin	0.06 to 8	1	2	0.5	−	−
Moxifloxacin	0.125 to 8	0.5	1	0.5	≤2	≥4
Levofloxacin	0.25 to 16	2	2	0.5	≤2	≥4
Tetracycline	0.015 to 0.25	0.125	0.25	8	≤1	≥2
Minocycline	≤0.0075 to 0.06	0.015	0.06	0.25	−	−
Doxycycline	≤0.0075 to 0.125	0.03	0.06	0.5	−	−
Erythromycin	0.25 to 2	1	1	1	≤8	≥16
Roxithromycin	0.125 to 1	0.25	0.5	0.25	−	−
Azithromycin	0.125 to 1	0.5	1	0.5	−	−
Josamycine	0.03 to 0.5	0.125	0.25	0.5	−	−
**Antimicrobial agents (μg/ml)^b^**						
Nemonoxacin	0.25 to >16	2	8	0.5	−	−
Moxifloxacin	0.125 to 8	4	4	0.5	≤2	≥4
Levofloxacin	0.5 to 16	4	8	0.5	≤2	≥4
Tetracycline	0.25 to >16	0.5	1	8	≤1	≥2
Minocycline	0.03 to 4	0.125	0.25	0.25	−	−
Doxycycline	0.06 to 8	0.125	0.25	0.5	−	−
Erythromycin	0.25 to 4	1	4	1	≤8	≥16
Roxithromycin	0.125 to 2	0.25	1	0.25	−	−
Azithromycin	0.125 to 4	0.5	2	0.5	−	−
Josamycine	0.015 to 1	0.125	0.25	0.5	−	−

*^*a*^Total of 64 *U. parvum*, including two fluoroquinolone-resistant organisms; ^*b*^Total of 13 *U. urealyticum*, including eight fluoroquinolone-resistant and one tetracycline-resistant organisms; ^*c*^Interpretive criteria from CLSI M43-A. S, susceptible; R, resistant.*

Meanwhile, we also collected eight fluoroquinolone-resistant isolates from among the 13 *U. urealyticum* strains; one was also resistant to the tetracyclines with *tet*(M). The nemonoxacin MICs of the eight fluoroquinolone-resistant isolates ranged from 2 to >16 μg/ml, while moxifloxacin and levofloxacin were 1–8 and 4–16 μg/ml, respectively. Seven fluoroquinolone-resistant isolates revealed the presence of the same mutation at position 248 (C-T) for *parC*, which leads to a substitution of Ser-83 by Leu in the parC protein. Another fluoroquinolone-resistant isolate existed, which had gene mutations at position 248 (C-T) and 310 (G-A) with a substitution of Ser-83 by Leu in the parC.

A detailed MIC distribution of 10 antibiotics against *Ureaplasma* spp. is shown in Supplementary Table 3.

### Nemonoxacin Is Bactericidal Against *M. pneumoniae*

In this study, the MBCs of nemonoxacin for *M. pneumoniae* were within twofold dilutions of the MIC, which indicates that it is bactericidal against *M. pneumoniae*. However, all MBCs for *M. hominis* and *Ureaplasma* spp. were three or more dilutions greater than their corresponding MICs, indicating a bacteriostatic effect.

## Discussion

A fluorine atom at position C-6 and various substitutions on the basic bicyclic ring structure produce fluoroquinolones, namely, norfloxacin, levofloxacin, ciprofloxacin, and many other drugs (Kocsis et al., 2016). Adding a fluorine at the sixth position can greatly improve the activity of fluoroquinolones compared with the former quinolones. Ciprofloxacin, levofloxacin, and moxifloxacin are currently the most commonly prescribed fluoroquinolones in medical practice, but their use is limited due to toxic side effects and an increasing number of resistant pathogens (Huang et al., 2015; Kocsis et al., 2016). Nemonoxacin may have increased antibacterial effectiveness and decreased frequency of toxic adverse effects due to its C-8-methoxy substituent on the quinolone ring and the lack of a fluorine residue (Barry et al., 2001; Andersson and MacGowan, 2003). In our research, nemonoxacin and moxifloxacin exhibited more potent antibacterial activities against *M. pneumoniae* than other fluoroquinolones, which may be attributed to their 8-methoxy structures that enable the targeting of both topoisomerases II (i.e., DNA gyrase) and IV (Siegert et al., 2000; Guo et al., 2012; Lai et al., 2014).

For many years, the empirical treatments of choice for *M. pneumoniae* infections were macrolides, especially for children, in whom tetracyclines and fluoroquinolones were limited because of possible toxicities (Waites et al., 2017b). Unfortunately, 92% (46/50) of macrolide resistance rates were detected in our study, which is similar to our previous study and other data from Asia. In the last 10 years, the macrolide resistance rates of *M. pneumoniae* have not changed in Shanghai, while in Europe and the United States, the resistance prevalence is substantially lower than that in Asia (Morozumi et al., 2008; Liu et al., 2009; Waites et al., 2017b). In addition, all macrolides resistant isolates in our study only had the one same mutation in 23S rRNA gene (A2063G), which is slightly different from previous studies that showed another mutation in the 2,064 position of the 23S rRNA gene (Pereyre et al., 2007; Morozumi et al., 2008; Xin et al., 2009). Josamycine, a 16-membered macrolide, differs from 14-membered macrolides by having extended C-5 sugar units, which can slow down the formation of the first peptide bond and inhibit formation of any further bonds. Methylation of A2058 (*Escherichia coli* numbering) in 23S rRNA is the classical resistance mechanism of 14-membered macrolides, but it does not have such a dramatic effect on 16-membered macrolides (Arsic et al., 2018). Indeed, josamycine had lower MICs for *M. pneumoniae* than did the 14-membered macrolides in our results. Until now, resistance to tetracyclines and fluoroquinolones in *M. pneumoniae* have never been reported (Waites et al., 2017b). No resistant strains to tetracyclines and fluoroquinolones were detected in our collected *M. pneumoniae* isolates. Between the macrolide sensitive and resistant strains, there was no evident difference in the nemonoxacin MICs. Therefore, mutations related to macrolides resistance may not influence the activity of nemonoxacin.

The 20 *M. hominis* isolates were not resistant to the tetracyclines we tested and had low josamycine MICs; however, 80% (16/20) of the isolates were resistant to the fluoroquinolones we tested. There are many previous studies that showed susceptibility of *M. hominis* to tetracyclines and fluoroquinolones, but few data support susceptibility to josamycine. In Korea, similarly, *M. hominis* isolates were susceptible to doxycycline and tetracycline (100 and 94.12%, respectively) (Choi et al., 2018). In France, Meygret et al. (2018) reported the resistance rate of *M. hominis* to tetracycline was 14.8% from 2010 to 2015, and Degrange et al. (2008) found the rate was 18.75% from 1999 to 2002. Almost all fluoroquinolone resistance in *M. hominis* was caused by *gyrA* and/or *parC* mutations in our research. In comparison with a parC mutation alone, it is interesting that an additional mutation in gyrA seems to increase the quinolone resistance by at least twofold, particularly for moxifloxacin and ciprofloxacin, which occurred in Meygret’s and Bebear’s articles as well (Bebear et al., 2000; Meygret et al., 2018). Like the *Ureaplasma* spp. strains, the higher nemonoxacin MICs in the fluoroquinolone-resistant strains may have gained resistance via mutations of the QRDRs, whereas those isolates without the mutations were susceptible.

In this study, we found only one *U. parvum* isolate that was resistant to tetracycline with *tet*(M), and no macrolide-resistant *Ureaplasma* spp. strains. In Switzerland, low rates of non-susceptibility were observed for clarithromycin (4.9%), erythromycin (1.9%), and azithromycin (1%), and there were no isolates that showed a reduced susceptibility to josamycine and tetracyclines, according to the IST 2 kit (Schneider et al., 2015). In the United States, all 250 *Ureaplasma* isolates detected were not resistant to erythromycin and had parallel MICs to azithromycin by broth microdilution, while one *U. parvum* isolate was resistant to tetracycline as well (Fernandez et al., 2016). The slight inconsistencies between various research studies might be due to different methodologies and interpretation criteria of susceptibility. Beeton et al. (2009b) found the tetracycline-susceptible strain (HPA6) was tetM positive, meanwhile Fernandez et al. (2016) also reported that one isolate with *tet*(M) in 10 *U. parvum* isolates was susceptible. However, no *tet*(M) element was found in the tetracycline-susceptible isolates in our study, which is in contrast to those mentioned above.

Regarding the detection rate, the fluoroquinolone-resistant rate and the resistance mechanism, a comparison of *U. urealyticum* with *U. parvum* highlights obvious differences in our results. Of the 77 *Ureaplasma* spp. strains we observed, we detected 13 *U. urealyticum* (16.88%) and 64 *U. parvum* (83.12%) strains. In other research studies, *U. urealyticum* was also isolated much less frequently than *U. parvum* from patients (Beeton et al., 2009b; Fernández et al., 2016). However, around a 61% (8/13) resistance rate of *U. urealyticum* to fluoroquinolones is far higher than *U. parvum*’s 3% (2/64) resistance rate. Of the fluoroquinolone-resistant *Ureaplasma* spp. strains, the portion of *U. urealyticum* was 80% (8/10). All fluoroquinolone-resistant *U. urealyticum* had a parC Ser83Leu mutation alone, whereas a gyrB Pro153Ser mutation was found in one of two fluoroquinolone-resistant *U. parvum* isolates. As previous studies, we also found that about 80% *U. urealyticum* was detected and the ParC Ser83Leu mutation was largely found in all fluoroquinolone-resistant *Ureaplasma* spp. (Beeton et al., 2009a). Compared with fluoroquinolone-susceptible isolates, the nemonoxacin MICs of fluoroquinolone-resistant strains had higher values in our results. Therefore, the mutations related to fluoroquinolones resistance in *Ureaplasma* spp. could affect the activity of nemonoxacin, which is similar to *Streptococcus pneumoniae*, *E. coli*, and methicillin-resistant *Staphylococcus aureus* (Lauderdale et al., 2010).

In summary, this report highlights the superior activity of nemonoxacin and moxifloxacin against *M. pneumoniae in vitro* in comparison with *Ureaplasma* spp. and *M. hominis.* For macrolide-resistant *M. pneumoniae* infections, 16-membered macrolides may be a great choice for children. According to different antibiotic susceptibilities, identifying *U. parvum* or *U. urealyticum* in clinical isolates could help clinicians choose appropriate drugs for treatment. For *U. urealyticum* infections especially, fluoroquinolones are not recommended because of their high resistance rate. Additionally, 16-membered macrolide and tetracycline can be used as treatments for *M. hominis* infections in China.

## Data Availability

All datasets generated for this study are included in the manuscript and/or the Supplementary Files.

## Ethics Statement

The strains, we used in this study, obtained from the biological sample and strains bank of the Institute of Antibiotics, Huashan Hospital, Shanghai. They come from the normal clinical testing. And then, they were stored in the strains bank. The ethics committee of Huashan Hospital authorized our study and written informed consent is not required. This study would not do harm to rights, benefits, and health of the subjects, and the privacy and personal identity information of the subjects will be protected.

## Author Contributions

YL and NW contributed to the design and implementation of the study. NW, WL, and YZ performed the experimental studies and analysis. All authors aided in the interpretation and outcomes of the experimental analyses, contributed to the revisions and editing of the manuscript, and read and approved the final version of the manuscript for submission. NW and WL wrote the first draft of the manuscript. YL and YZ extensively edited the subsequent drafts of the manuscript.

## Conflict of Interest Statement

The authors declare that the research was conducted in the absence of any commercial or financial relationships that could be construed as a potential conflict of interest.
