# Supplementary material for: In vitro Activities of Nemonoxacin and Other Antimicrobial Agents Against Human Mycoplasma and Ureaplasmas Isolates and Their Defined Resistance Mechanisms
Source: Front Microbiol. 2019 Aug 13;10:1890. doi: 10.3389/fmicb.2019.01890 (PMC6700270; doi:10.3389/fmicb.2019.01890)
Supplement: Supplementary file 1 [file Table_1.DOC]

Supplementary table 1

Distribution of 10 antimicrobial MICs for 50 *M. pneumoniae* clinical strains

| **Antibiotics** | **MIC** (μg/ml, strain No.) | | | | | | | | | | | | | | | | |
| --- | --- | --- | --- | --- | --- | --- | --- | --- | --- | --- | --- | --- | --- | --- | --- | --- | --- |
|  | **≤0.00375** | **0.0075** | **0.015** | **0.03** | **0.06** | **0.125** | **0.25** | **0.5** | **1** | **2** | **4** | **8** | **16** | **32** | **64** | **128** | **＞128** |
| Nemonoxacin |  |  |  | 1 | 2 | 22 | 25 |  |  |  |  |  |  |  |  |  |  |
| Moxifloxacin |  |  | 1 |  | 2 | 28 | 19 |  |  |  |  |  |  |  |  |  |  |
| Levofloxacin |  |  |  | 1 |  |  | 10 | 34 | 5 |  |  |  |  |  |  |  |  |
| Ciprofloxacin |  |  |  | 1 |  |  | 1 | 19 | 21 | 8 |  |  |  |  |  |  |  |
| Tetraiycline |  |  |  |  | 3 | 8 | 22 | 13 | 3 | 1 |  |  |  |  |  |  |  |
| Minocycline |  |  |  | 1 | 3 | 9 | 27 | 9 | 1 |  |  |  |  |  |  |  |  |
| Doxycycline |  |  | 1 | 1 | 7 | 26 | 9 | 2 | 4 |  |  |  |  |  |  |  |  |
| Erythromycin |  | 2 | 1 |  | 1 |  |  |  |  |  |  |  |  |  | 2 | 16 | 28 |
| Roxithromycin | 1 | 2 | 1 |  |  |  |  |  |  |  |  |  |  | 7 | 17 | 9 | 13 |
| Azitheomycin | 4 |  |  |  |  |  |  |  |  | 1 | 10 | 9 | 17 | 6 | 3 |  |  |
| Josamycine | 2 | 1 |  |  |  | 1 |  |  | 9 | 17 | 15 | 4 |  | 1 |  |  |  |

Supplementary table 2

Distribution of 10 antimicrobial MICs for 20 *M. hominis* clinical strains

| **Antibiotics** | **MIC** (μg/ml, strain No.) | | | | | | | | | | | | | | | | |
| --- | --- | --- | --- | --- | --- | --- | --- | --- | --- | --- | --- | --- | --- | --- | --- | --- | --- |
|  | **≤0.00375** | **0.0075** | **0.015** | **0.03** | **0.06** | **0.125** | **0.25** | **0.5** | **1** | **2** | **4** | **8** | **16** | **32** | **64** | **128** | **＞128** |
| Nemonoxacin |  |  |  |  |  |  | 3 | 1 | 2 | 7 | 4 | 3 |  |  |  |  |  |
| Moxifloxacin |  |  |  |  | 3 | 5 | 1 | 1 |  | 2 | 4 | 4 |  |  |  |  |  |
| Levofloxacin |  |  |  |  |  |  | 3 | 1 |  | 1 | 8 | 6 | 1 |  |  |  |  |
| Ciprofloxacin |  |  |  |  |  |  |  | 1 | 3 | 4 | 2 | 5 | 3 | 2 |  |  |  |
| Tetraiycline |  |  |  | 3 | 15 | 2 |  |  |  |  |  |  |  |  |  |  |  |
| Minocycline |  | 18 | 2 |  |  |  |  |  |  |  |  |  |  |  |  |  |  |
| Doxycycline |  | 18 | 1 | 1 |  |  |  |  |  |  |  |  |  |  |  |  |  |
| Erythromycin |  |  |  |  |  |  |  |  |  |  |  |  |  |  |  |  | 20 |
| Roxithromycin |  |  |  |  |  |  |  |  |  |  |  |  |  |  |  |  | 20 |
| Azitheomycin |  |  |  |  |  |  |  |  |  |  |  |  |  |  | 1 | 3 | 16 |
| Josamycine |  |  |  |  |  | 1 | 5 | 13 | 1 |  |  |  |  |  |  |  |  |

Supplementary table 3

Distribution of 10 antimicrobial MICs for 77 *Ureaplasma species* clinical strains

| **Antibiotics** | **MIC** (μg/ml, strain No.) | | | | | | | | | | | | |
| --- | --- | --- | --- | --- | --- | --- | --- | --- | --- | --- | --- | --- | --- |
|  | **≤0.0075** | **0.015** | **0.03** | **0.06** | **0.125** | **0.25** | **0.5** | **1** | **2** | **4** | **8** | **16** | **＞16** |
| Nemonoxacin |  |  |  | 1 |  | 3 | 18 | 13 | 33 | 2 | 6 |  | 1 |
| Moxifloxacin |  |  |  |  | 3 | 18 | 23 | 24 | 2 | 5 | 2 |  |  |
| Levofloxacin |  |  |  |  |  | 1 | 13 | 14 | 45 | 2 | 2 |  |  |
| Tetraiycline |  | 1 | 2 | 23 | 28 | 12 | 6 | 4 |  |  |  |  | 1 |
| Minocycline | 8 | 30 | 21 | 11 | 3 | 3 |  |  |  | 1 |  |  |  |
| Doxycycline | 3 | 14 | 30 | 19 | 6 | 4 |  |  |  |  | 1 |  |  |
| Erythromycin |  |  |  |  |  | 13 | 18 | 42 | 2 | 2 |  |  |  |
| Roxithromycin |  |  |  |  | 14 | 44 | 15 | 3 | 1 |  |  |  |  |
| Azitheomycin |  |  |  |  | 10 | 23 | 29 | 13 | 1 | 1 |  |  |  |
| Josamycine |  | 1 | 3 | 20 | 29 | 18 | 5 | 1 |  |  |  |  |  |
